# Monocyte to high-density lipoprotein and apolipoprotein A1 ratios are associated with bone homeostasis imbalance caused by chronic inflammation in postmenopausal women with type 2 diabetes mellitus

**DOI:** 10.3389/fphar.2022.1062999

**Published:** 2022-11-07

**Authors:** Rong Huang, Yang Chen, Mei Tu, Wei Wang

**Affiliations:** Department of Endocrinology, Longyan First Affiliated Hospital of Fujian Medical University, Longyan, Fujian, China

**Keywords:** chronic inflammation, monocyte to high-density lipoprotein ratio, monocyte to apolipoprotein A1 ratio, bone homeostasis imbalance, type 2 diabetes mellitus

## Abstract

**Objective:** Emerging evidences demonstrated that chronic inflammation can influence bone metabolism in type 2 diabetes mellitus (T2DM), leading to bone homeostasis imbalance. The aim of this study was to assess the correlations between novel pro-inflammatory indexes like monocyte to high-density lipoprotein (MHR), apolipoprotein A1 (MAR) ratios and bone mineral density (BMD), bone turnover markers in Chinese postmenopausal women with T2DM.

**Method:** In this study, a total of 619 participants with complete data were included in the final analysis. Demographic and anthropometric information was collected. Biochemical parameters and bone turnover markers were determined by standard methods. BMD was measured by dual-energy x-ray absorptiometry. Correlation analysis and regression models were conducted to assess the associations between MHR, MAR and bone turnover markers, BMD. Multiple binomial logistic regression model was used to estimate the independent variables of MHR and MAR for osteoporosis.

**Results:** Overall, the prevalence of osteoporosis was 38.3%. MHR and MAR were significantly correlated with C-terminal cross linking of type I collagen (β-CTX), L1-L4, femoral neck BMD and T scores. These correlations remained significant after adjustment for other confounding factors. Meanwhile, MHR and MAR were also significantly associated with higher odds of osteoporosis, the odds ratios (ORs) (95%CI) were 1.88 (1.49–2.38) and 2.30 (1.72–3.09) respectively. Furthermore, MHR and MAR seemed to have a good identifying value for osteoporosis. The area under the curve of MHR and MAR identifying osteoporosis were 0.791 (95% CI: 0.753–0.828) and 0.843 (95% CI: 0.809–0.877) respectively (*p* < 0.001). The optimal cut-off values of MHR and MAR were 4.53 × 10^8^/mmol (sensitivity: 60.8%, specificity: 85.9%) and 4.74 × 10^8^/g (sensitivity: 71.7%, specificity: 89.3%) respectively.

**Conclusion:** MHR and MAR were significantly associated with osteoporosis. These two novel pro-inflammatory indexes may be ideal markers to reflect bone homeostasis imbalance caused by chronic inflammation in Chinese postmenopausal women with T2DM.

## Introduction

Bones are important tissues belonged to dynamic and metabolically active tissues that can support the body and protect the internal organs. Bones experience a continuous cycle of bone remodeling and depend on the activity of osteoblasts and osteoclasts on the surface of the bones to keep balance between bone formation and resorption (Charles Julia and Aliprantis Antonios, 2014). This balance ensures bones can adapt to mechanical loads changes and minor damages. To our concerns, this balance can be easily upset by some diseases. Type 2 diabetes (T2DM) is a kind of metabolic and chronic inflammatory disease characterized by chronic hyperglycemia that can break the balance between bone formation and resorption. Epidemiological investigations found that T2DM can increase the risk of osteoporosis and bone fragility fractures, both of them are rapidly increasing (Bonds Denise et al., 2006; Li et al., 2019; Thong et al., 2021). Elevated advance glycation end products (AGEs), increased insulin resistance, dyslipidemia, obesity, and chronic inflammatory statues caused by T2DM are the main underlying mechanisms for T2DM to increase the risk of osteoporosis and bone fragility fractures (McCarthy Antonio et al., 2004; Shapses Sue and Sukumar, 2012; Conte et al., 2018; Yin et al., 2019; Maruyama et al., 2020). Among these underlying mechanisms, chronic inflammation triggered by metabolic disorders plays important roles in bone metabolism, it can directly influence bone formation and resorption, leading to bone mass loss and osteoporosis (Adamopoulos Iannis, 2018).

Monocyte are important innate immune cells produced in bone marrow and accumulated in circulatory system before migrating and differentiating into macrophages, it play functional roles in immune defense, chronic inflammation, and tissue remodeling (Orozco Susana et al., 2021). These biological features provide a basis for monocyte involved in the bone homeostasis imbalance caused by chronic inflammation. Besides dyslipidemia has the well recognized effects in promoting arteriosclerosis, more evidences also found that dyslipidemia was also associated with increased oxidative stress and systemic inflammation, leading to increased osteoclastic activity and decreased bone formation (Pelton et al., 2012). T2DM are often accompanied with dyslipidemia like increased low-density lipoprotein cholesterol (LDL-c) and decreased high-density lipoprotein cholesterol (HDL-c) and apolipoprotein A1 (APOA1). Recently, the ratios of monocyte to HDL-c (MHR) or APOA1 (MAR) have been considered as pro-inflammatory indexes and were reported to have good predictive values for chronic low inflammatory diseases like peripheral artery disease (Selvaggio et al., 2020), polycystic ovarian syndrome (Kałużna et al., 2020) and nonalcoholic fatty liver disease (Wang et al., 2022). Based on the above studies, we can be hypothesized that MHR and MAR may be able to reflect bone homeostasis imbalance cause by chronic inflammation in T2DM. To prove this hypothesis, this cross-sectional study was aimed to assess the correlations between MHR, MAR and BMD, bone turnover markers in Chinese postmenopausal women with T2DM.

## Study design and methods

### Study design and participants

**FIGURE 1:**
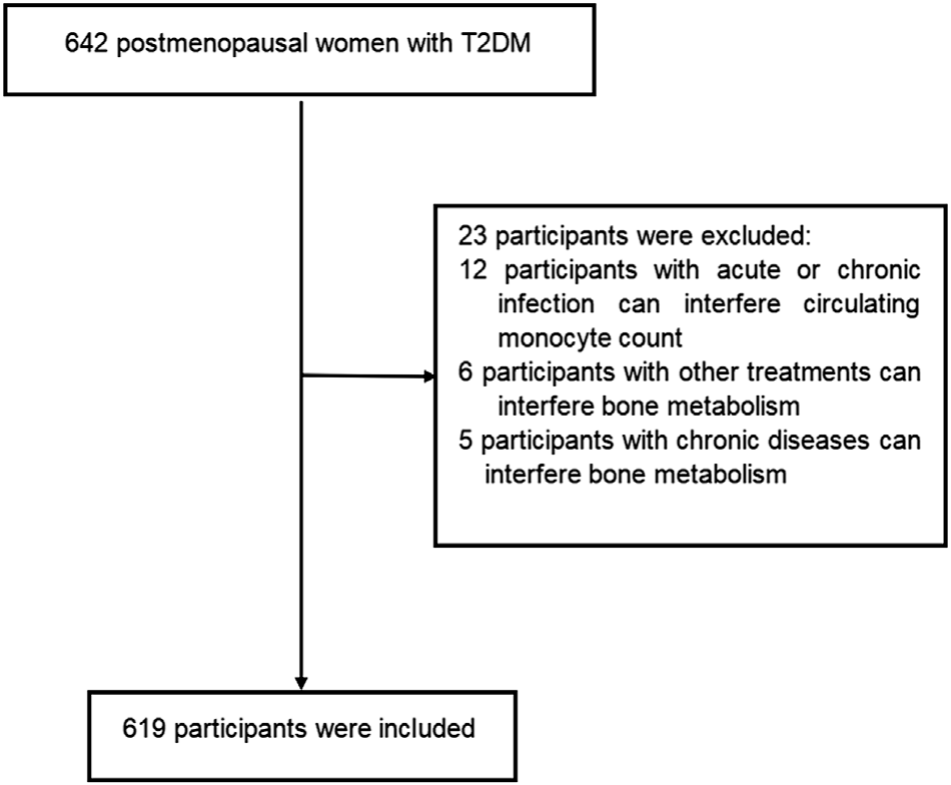
Flow diagram of the participants excluded and included in this study.

This cross-sectional study was conducted with postmenopausal women with T2DM from the Department of Endocrinology at the Longyan First Affiliated Hospital of Fujian Medical University who fulfilled the study criteria between January 2022 and June 2022. Participants were diagnosed with T2DM according to the World Health Organization criteria (2019 edition): 1) fasting plasma glucose ≥126 mg/dl or 2 h postprandial ≥200 mg/dl during oral glucose tolerance test or HbA1C ≥ 6.5% or participants with classic symptoms of hyperglycemia or hyperglycemic crisis with random plasma glucose ≥200 mg/dl. 2) with diabetic autoimmune antibodies negative and exclude other specific types of diabetes. Participants with twelve consecutive months of amenorrhea was considered as postmenopausal women. Participants were excluded if they met the following criteria: 1) presence of acute or chronic infection, obvious liver or renal dysfunction, anemia, hemolytic diseases and bleeding that can interfere circulating monocyte count. 2) treatment with medications can interfere circulating monocyte count. 3)history of chronic diseases can interfere bone metabolism (i.e.,renal, hepatic, cardiac, thyroid and rheumatic diseases). 4) current or prior use of drugs can interfere bone metabolism (i.e., glucocorticoids, anti-resorptive drugs, hormonal replacement therapy, calcium or vitamin D supplementation, anti-osteoporosis therapy, thiazolidinediones, and urate-lowering therapy). The prevalence of osteoporosis is about 30%–40% in postmenopausal women with T2DM. In present study, we estimated the sample size according to the requirement of multiple binomial logistic regression model.12–15 variables may be put into the logistic regression model. Based on the principle of 5–10 events per variable, we planed sample size is 500–600 participants. Overall, a total of 642 participants were screened. Among them, 619 participants met the inclusion and exclusion criteria were enrolled into this study. The flow diagram of excluded and included participants was presented in Figure 1. All procedures were conducted in accordance with Declaration of Helsinki. This study was approved by the ethical committee of Longyan First Affiliated Hospital of Fujian Medical University (LY-2021–072). All participants provided informed consent.

### Anthropometric and laboratory assessments

Demographic information was collected by trained interviewers through a standard questionnaire. A review of medical records and laboratory data were also conducted to obtain demographic information, including age, diabetes duration, history of disease, current or prior use of drugs, smoking, drinking, physic activity and menopausal status. Participants have any waking behaviors characterized by an energy expenditure≤1.5 metabolic equivalents (e.g., watching television, reading, or reclining) were considered to have sedentary behavior (DiPietro et al., 2020). Participants smoke more than 4 cigarettes a week for at least 6 months continually or accumulative was considered as smoking (Organization, 1998). Participants drink more than once a year was considered as drinking (GBD 2016, 2018). Anthropometric examination was conducted by the research nurses, including height, weight and blood pressure (BP). Participants wear hospital gowns and had bare feet. BMI was calculated as the weight divided by the square of height (kg/m^2^). Systolic and diastolic blood pressure (SBP and DBP) were measured by an electronic sphygmomanometer with an appropriate cuff size after the participants taking a rest for more than 5 min. Participants with anti-hypertensive therapy or three times measurements of SBP≥140 mmHg or DBP ≥90 mmHg on different days was considered to have hypertension.

Laboratory assessments were conducted according to standard methods using fasting venous blood samples that were taken between 8 and 9 a.m. after fasting overnight. Serum levels of the following variables were determined: creatinine, alanine aminotransferase (ALT), uric acid (UA), fasting blood glucose (FBG), serum insulin, HbA1c, diabetic autoimmune antibodies (GADA, IAA, and ICA), HDL-c, low density lipoprotein (LDL-c), triglycerides (TGs), APOA1, calcium, phosphorous, alkaline phosphatase (ALP), high sensitivity C-reactive protein (hs-CRP), complete blood count, thyroid stimulating hormone (TSH) and bone turnover markers like osteocalcin (OC), *β*-cross-linked C-telopeptide of type I collagen (β-CTX), intact parathyroid hormone (iPTH) and 25 hydroxyvitamin D (25-OH-D). Biochemical indexes were measured by an auto-biochemical analyzer (Roche Diagnostics Corporation). ApoA1 levels were measured by the polyethylene glycol-enhanced immunoturbidimetric assay (Maker, Chengdu, China). HbA1c was evaluated by high performance liquid chromatography with a D10 set (Bio-RAD). Complete blood count were measured by Coulter LH 780 Analyzer (Beckman Coulter Ireland, Galway, Ireland). Bone turnover markers were measured by electro-chemiluminescence immunoassay method (Roche Diagnostics GmbH, Germany). Homeostasis model assessment (HOMA-IR) was calculated with the formula: fasting serum insulin (µU/ml) x fasting plasma glucose (mmol/L)/22.5 (Bonora et al., 2000). The monocyte count divided by HDL-c or APOA1 level were calculated as MHR or MAR.

### Assessment of bone mineral density

Participants were performed with BMD examination by dual-energy X-ray absorptiometry (Hologic, Marlborough, MA, United States) in a supine position. The BMD measurement areas consist of lumbar spine (L1-L4), total hip and femoral neck. The densitometry scanning was performed by experienced radiographers who were blinded to clinical information. T-score was calculated according to Hologic densitometry reference value. Longitudinal quality control checks were performed daily using whole-body and L1-L4 lumbar spine phantom provided by the manufacturer. Cross-calibration was performed weekly to monitor variations between the systems. The precision error was 1.0% for the BMD measurement. Postmenopausal women with T score ≤ -2.5 or a history of bone fragility fractures was considered to have osteoporosis.

### Statistical analysis

Data were analyzed by using the SPSS 23.0 software (SPSS Inc. IBM). Descriptive data were expressed as means ± standard deviation (SD). Discrete variables were summarized in frequency tables (N, %). Statistical differences among groups were performed with one-way analysis of variance (ANOVA) followed by Turkey test for multiple comparisons. The chi-squared (χ2) test or Fisher exact test were used for comparison of categorical variables. The correlations between MAR, MHR and bone turnover markers, BMD were performed by Pearson or Spearman correlation analysis. Multiple regression analysis was used to estimate independent associations between MHR, MAR and bone turnover markers, BMD after adjusting for potential confounding factors. Multiple binomial logistic regression model was used to estimate the independent variables of MHR and MAR for osteoporosis after adjusting for other covariates. The receiver operating characteristic (ROC) curves was used to assess the identifying value of MHR and MAR for osteoporosis. Optimal cut-off value was based on the greatest value of the Youden’s index. A two-tailed value of *p* < 0.05 was considered statistically significant.

## Results

Overall, a total of 619 participants with complete data were included in the final analysis, whose mean age was 55.8 ± 6.1 years and diabetes duration was 7.9 ± 2.4 years. Clinical and laboratory characteristics of participants based on tertiles of MHR and MAR were summarized in Table 1. There were no significant differences in level of age, diabetes duration, TC, LDL-c, creatinine, ALT and the percentage of participants with smoking, drinking and sedentary behavior across the MHR and MAR tertiles (*p* > 0.05). Increasing trends were observed in BMI, TG, UA, HOMA-IR and monocyte count across the MHR and MAR tertile (*p <* 0.05). Decreasing trends were also observed in HDL-c and APOA1 across the MHR and MAR tertile (*p <* 0.05). Furthermore, participants in higher tertile of MHR and MAR groups showed higher prevalence of hypertension (*p <* 0.05). Bone turnover markers and BMD of participants based on tertiles of MHR and MAR were presented in Table 2. The prevalence of osteoporosis was 38.3%.As we had expected, increasing trend was observed in *β*-CTX. Decreasing trends were also observed in L1-L4 BMD, L1-L4 T-score, femoral neck BMD and femoral neck T-score (*p <* 0.05). In addition, participants in higher tertile of MHR and MAR groups showed higher prevalence of osteoporosis (*p <* 0.05).

**TABLE 1:** Clinical and laboratory characteristics of participants based on tertiles of MHR (10^8^/mmol) and MAR (10^8^/g).

Variable	Tertiles of MHR			*P*	Tertiles of MAR			*P*
	T1	T2	T3		T1	T2	T3	
Age (year)	55.7 ± 6.3	56.4 ± 6.8	55.4 ± 5.1	0.230	55.1 ± 7.1	56.1 ± 5.3	56.3 ± 5.8	0.123
Duration (year)	7.8 ± 2.3	8.1 ± 2.9	8.1 ± 2.4	0.407	7.9 ± 2.2	8.1 ± 2.7	8.0 ± 2.3	0.482
BMI(kg/m^2^)	23.4 ± 2.6^ab^	24.1 ± 2.5^ac^	24.7 ± 3.2^bc^	<0.001	22.4 ± 2.6^ab^	24.2 ± 2.4^ac^	25.6 ± 2.6^bc^	<0.001
HbA1c (%)	9.0 ± 1.3	8.8 ± 1.2	8.9 ± 1.0	0.218	9.0 ± 1.2	8.8 ± 1.2	8.9 ± 1.1	0.392
TG (mmol/L)	1.3 ± 0.9^ab^	1.9 ± 0.7^ac^	3.3 ± 1.5^bc^	<0.001	1.3 ± 0.7^ab^	2.0 ± 0.8^ac^	3.1 ± 1.3^bc^	<0.001
TC (mmol/L)	5.1 ± 1.2	5.0 ± 1.2	5.1 ± 1.1	0.408	5.1 ± 1.3	5.1 ± 1.2	5.0 ± 1.1	0.503
HDL-c (mmol/L)	1.2 ± 0.2^ab^	1.1 ± 0.2^ac^	0.9 ± 0.2^bc^	<0.001	1.3 ± 0.2^ab^	1.0 ± 0.2^ac^	0.9 ± 0.1^bc^	<0.001
LDL-c (mmol/L)	3.6 ± 1.0	3.5 ± 1.0	3.6 ± 1.0	0.378	3.5 ± 1.0	3.6 ± 0.9	3.6 ± 1.0	0.503
APOA1 (g/L)	1.13 ± 0.21^b^	1.11 ± 0.17^c^	0.98 ± 0.16^bc^	<0.001	1.11 ± 0.23^b^	1.10 ± 0.22^c^	1.01 ± 0.20^bc^	<0.001
Monocyte (10^8^/L)	3.5 ± 0.7^ab^	4.3 ± 0.7^ac^	4.8 ± 0.8^bc^	<0.001	3.6 ± 0.6^ab^	4.3 ± 0.6^ac^	4.7 ± 0.7^bc^	<0.001
UA (umol/L)	323 ± 72^ab^	353 ± 77^ac^	373 ± 99^bc^	<0.001	296 ± 69^ab^	369 ± 73^ac^	396 ± 87^bc^	<0.001
Creatinine (umol/L)	69.2 ± 13.7	70.7 ± 12.9	71.1 ± 12.2	0.315	69.9 ± 13.0	70.1 ± 13.6	71.0 ± 12.2	0.686
ALT (IU/L)	34.2 ± 8.7	33.9 ± 9.2	33.9 ± 8.6	0.917	33.8 ± 9.1	34.3 ± 9.0	33.9 ± 8.5	0.823
HOMA-IR	9.1 ± 4.8^ab^	10.8 ± 5.3^ac^	12.4 ± 5.9^bc^	<0.001	7.6 ± 4.7^ab^	11.2 ± 4.7^ac^	13.6 ± 5.4^bc^	<0.001
Hypertension, n (%)	61 (29.5)^b^	72 (35.0)^c^	124 (60.2)^bc^	<0.001	42 (20.3)^ab^	71 (33.8)^ac^	143 (70.8)^bc^	<0.001
Smoking,n (%)	4 (1.9)	7 (3.4)	7 (3.4)	0.592	5 (2.4)	7 (3.4)	6 (3.0)	0.854
Drinking,n (%)	35 (16.9)	33 (16.0)	33 (16.0)	0.961	32 (15.5)	40 (19.0)	29 (14.4)	0.401
Sedentary behavior,n (%)	52 (25.1)	63 (30.6)	65 (31.6)	0.300	56 (27.1)	58 (27.6)	66 (32.7)	0.388

BMI, body mass index; HbA1c, Glycated hemoglobin. UA, uric acid. TG, triglyceride. TC, total cholesterol. HDL-c, high-density lipoprotein cholesterol. LDL-c, Low density lipoprotein cholesterol. SBP: Systolic blood pressure. DBP: Diastolic blood pressure. HOMR-IR:Homeostasis model assessment insulin resistance. ^a^P < 0.05: T1 vs. T2. ^b^P <0.05: T1 vs. T3. ^c^P < 0.05: T2 vsT3.

**TABLE 2:** Bone turnover markers and bone mineral density of participants based on tertiles of MHR (10^8^/mmol) and MAR (10^8^/g).

Variable	Tertiles of MHR			*P*	Tertiles of MAR			P
	T1	T2	T3		T1	T2	T3	
OC(ng/ml)	14.8 ± 6.9	15.6 ± 6.8	15.3 ± 7.1	0.503	15.1 ± 6.7	15.4 ± 7.0	15.4 ± 7.2	0.872
β-CTX (ng/ml)	0.42 ± 0.19^ab^	0.47 ± 0.18^ac^	0.52 ± 0.16^bc^	<0.001	0.43 ± 0.19^ab^	0.47 ± 0.18^ac^	0.51 ± 0.16^bc^	<0.001
25-OH-D (nmol/L)	66.6 ± 11.6	67.0 ± 12.0	68.9 ± 13.1	0.121	67.7 ± 12.3	67.3 ± 12.0	67.4 ± 12.7	0.939
iPTH(ng/L)	35.1 ± 12.9	34.0 ± 14.0	32.9 ± 10.8	0.229	33.2 ± 12.8	34.6 ± 13.0	34.2 ± 12.6	0.425
ALP(IU/L)	80.5 ± 20.9	81.1 ± 18.2	80.0 ± 17.9	0.839	79.0 ± 20.0	81.0 ± 18.2	81.6 ± 18.9	0.351
Calcium (mmol/L)	2.32 ± 0.10	2.33 ± 0.10	2.31 ± 0.12	0.365	2.32 ± 0.11	2.32 ± 0.11	2.31 ± 0.11	0.793
Phosphorous (mmol/L)	1.22 ± 0.18	1.22 ± 0.17	1.22 ± 0.17	0.951	1.23 ± 0.19	1.22 ± 0.14	1.21 ± 0.18	0.251
L1-L4 BMD(g/cm^3^)	0.94 ± 0.13^ab^	0.88 ± 0.10^ac^	0.83 ± 0.09^bc^	<0.001	0.95 ± 0.13^ab^	0.88 ± 0.10^ac^	0.82 ± 0.09^bc^	<0.001
L1-L4 T-score	−1.3 ± 1.1^ab^	−1.8 ± 0.8^ac^	−2.2 ± 0.8^bc^	<0.001	−1.2 ± 1.1^ab^	−1.8 ± 0.7^ac^	−2.3 ± 0.7^bc^	<0.001
Hip BMD(g/cm^3^)	0.82 ± 0.09	0.84 ± 0.10	0.83 ± 0.12	0.543	0.83 ± 0.12	0.83 ± 0.07	0.83 ± 0.13	0.876
Hip T-score	−1.2 ± 0.6	−1.1 ± 0.7	−1.2 ± 0.7	0.617	−1.2 ± 0.5	−1.2 ± 0.6	−1.2 ± 0.8	0.894
Femoral neck BMD(g/cm^3^)	0.76 ± 0.24^ab^	0.70 ± 0.26^ac^	0.65 ± 0.13^bc^	<0.001	0.77 ± 0.24^ab^	0.71 ± 0.26^ac^	0.63 ± 0.13^bc^	<0.001
Femoral neck T-score	−0.9 ± 0.5^ab^	−1.3 ± 0.7^ac^	−1.6 ± 0.8^bc^	<0.001	−0.8 ± 0.5^ab^	−1.2 ± 0.7^ac^	−1.7 ± 0.8^bc^	<0.001
Osteoporosis,n (%)	23 (11.1)	68 (33.0)	146 (70.9)	<0.001	22 (10.6)	49 (23.3)	166 (82.2)	<0.001

OC, steocalcin. *β*-CTX, *β*-cross-linked C-telopeptide of type I collagen (β-CTX). 25-OH-D: 25 hydroxyvitamin D. iPTH:intact parathyroid hormone. ALP, alkaline phosphatase. BMD, bone mineral density. ^a^P<0.05: T1 vs. T2. ^b^P<0.05: T1 vs. T3. ^c^P<0.05: T2 vsT3.

**FIGURE 2:**
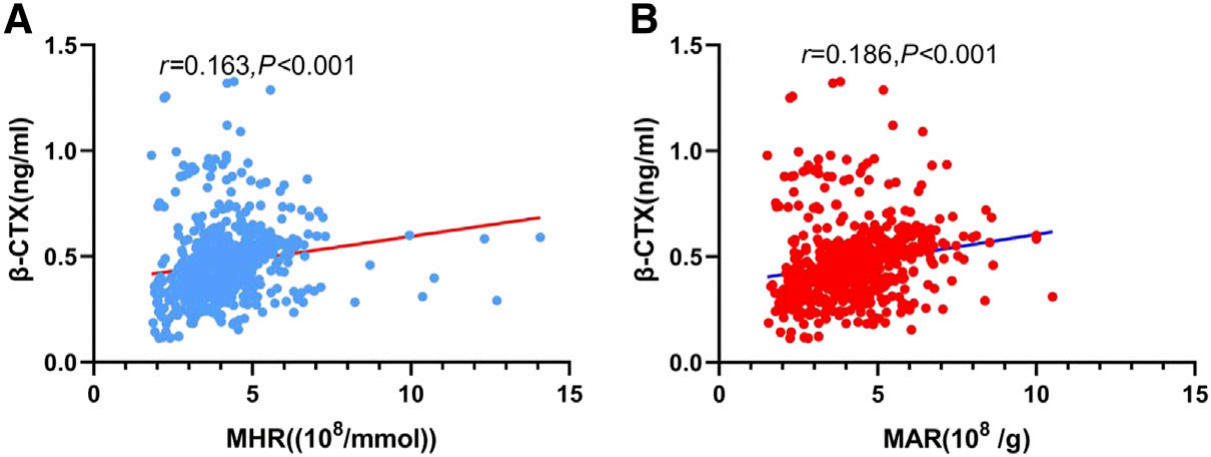
Correlation analysis showing positive correlation between *β*-CTX and MHR **(A)**, MAR **(B)**.

The main correlations between MHR, MAR and bone turnover markers, bone mineral density were presented in Table 3. The results showed that *β*-CTX was positively correlated with MHR (Figure 2A) and MAR (Figure 2B). Furthermore, MHR and MAR were also negatively correlated with L1-L4 BMD, L1-L4 T-score, femoral neck BMD and femoral neck T-score (*p <* 0.05). Whereas no significant associations were observed between MHR, MAR and OC, ALP, 25-OH-D, iPTH, calcium, phosphorous, hip BMD and T-score. To determine independent variables of MHR and MAR for *β*-CTX, L1-L4 BMD, L1-L4 T-score, femoral neck BMD and femoral neck T-score, multiple linear regression analysis was also performed (Table 4). The results showed that MHR and MAR were positively correlated with *β*-CTX and were negatively correlated with L1-L4 BMD, L1-L4 T-score, femoral neck BMD and femoral neck T-score after adjustment for clinical variables like age, diabetes duration, BMI, TC, TG, LDL-c, creatinine, ALT, UA, hypertension, smoking, drinking and sedentary behavior (Model 1). Furthermore, MHR and MAR remain significantly correlated with *β*-CTX, L1-L4 BMD, L1-L4 T-score, femoral neck BMD and femoral neck T-score after additional adjustment for bone turnover markers likes OC, 25-OH-D, ALP, iPTH, calcium, phosphorous (Model 2) and *β*-CTX (Model 3).

**TABLE 3:** Correlations between MHR, MAR and bone turnover markers, bone mineral density.

Variable	MHR		MAR	
	R	P	R	P
OC(ng/ml)	0.018	0.660	0.015	0.714
β-CTX (ng/ml)	0.163	<0.001	0.186	<0.001
25-OH-D (nmol/L)	0.072	0.079	−0.044	0.271
iPTH(ng/L)	−0.073	0.072	0.025	0.533
ALP(IU/L)	0.007	0.867	0.058	0.234
Calcium (mmol/L)	0.023	0.565	0.002	0.959
Phosphorous (mmol/L)	0.043	0.283	−0.010	0.795
L1-L4 BMD(g/cm^3^)	−0.285	<0.001	−0.379	<0.001
L1-L4 T-score	−0.318	<0.001	−0.415	<0.001
Hip BMD(g/cm^3^)	0.016	0.692	0.005	0.911
Hip T-score	0.011	0.782	0.006	0.927
Femoral neck BMD(g/cm^3^)	−0.321	<0.001	−0.298	<0.001
Femoral neck T-score	−0.343	<0.001	−0.318	<0.001

OC, osteocalcin. β-CTX, *β*-cross-linked C-telopeptide of type I collagen (β-CTX). 25-OH-D: 25 hydroxyvitamin D. iPTH:intact parathyroid hormone. ALP, alkaline phosphatase. BMD, bone mineral density.

**TABLE 4:** Multivariate linear regression analysis of associations between MHR, MAR and bone turnover markers, bone mineral density.

Variable	MHR		MAR	
	*β*	*P*	*β*	*P*
β-CTX (ng/ml)				
Model 1	0.132	0.002	0.279	<0.001
Model 2	0.148	<0.001	0.275	<0.001
L1-L4 BMD(g/cm^3^)				
Model 1	−0.241	<0.001	−0.394	<0.001
Model 3	−0.231	<0.001	−0.326	<0.001
L1-L4 T-score				
Model 1	−0.231	<0.001	−0.375	<0.001
Model 3	−0.220	<0.001	−0.294	<0.001
Femoral neck BMD(g/cm^3^)				
Model 1	−0.196	<0.001	−0.303	<0.001
Model 3	−0.177	<0.001	−0.249	<0.001
Femoral neck T-score				
Model 1	−0.203	<0.001	−0.291	<0.001
Model 3	−0.186	<0.001	−0.256	<0.001

Model 1:Adjusted for axge, diabetes duration,A1c, BMI, TG, HDL-c, LDL-c, creatinine, ALT,UA,HOMA-IR,hypertension, sedentary behavior, smoking and drinking. Model 2:Additional adjustment for bone turnover markers, such as OC,25-OH-D,iPTH,ALP, calcium and phosphorous based on Model 1. Model 3: Additional adjustment for *β*-CTX based on Model 2.

Binomial logistic regression analysis was also conducted to assess the independent variables of MHR and MAR for osteoporosis. As shown in Table 5, the MHR and MAR were shown to be independently associated with osteoporosis after adjustment for age, diabetes duration, hypertension, sedentary behavior, smoking and drinking (Model 4), the ORs (95%CI) were 2.82 (2.28–3.49) and 3.56 (2.83–4.49) respectively. Significant associations between MHR, MAR and osteoporosis were also observed after further adjustment for BMI, HbA1c, BMI, TG, HDL-c, LDL-c, creatinine, ALT, UA, HOMA-IR (Model 5), the ORs (95%CI) were 1.92 (1.52–2.44) and 2.68 (2.03–3.55) respectively. In addition, the ORs remain significant after further adjustment for bone turnover markers like OC, *β*-CTX, 25-OH-D, iPTH, ALP, calcium and phosphorous (Model 6), the ORs (95%CI) were 1.88 (1.49–2.38) and 2.30 (1.72–3.09) respectively.

**TABLE 5:** Binomial Logistic Regression Analysis adjusted ORs (95% CIs) for the associations between MHR, MAR and the risk of osteoporosis.

Models	MHR		MAR	
	OR (95%CI)	P	OR (95%CI)	P
Model 4	2.82 (2.28–3.49)	<0.001	3.56 (2.83–4.49)	<0.001
Model 5	1.92 (1.52–2.44)	<0.001	2.68 (2.03–3.55)	<0.001
Model 6	1.88 (1.49–2.38)	<0.001	2.30 (1.72–3.09)	<0.001

Model 4:Adjustment for age, diabetes duration, hypertension, sedentary behavior, smoking and drinking. Model5:Additionally adjustment for BMI,HbA1c, BMI, TG, HDL-c, LDL-c, creatinine, ALT,UA,HOMA-IR.Model 6:Additional adjustment for bone turnover markers, such as OC, *β*-CTX, 25-OH-D,i PTH, ALP, calcium and phosphorous based on Model 5. MHR:monocyte to HDL-c ratio. MAR:monocyte to ApoA1 ratio.

**FIGURE 3:**
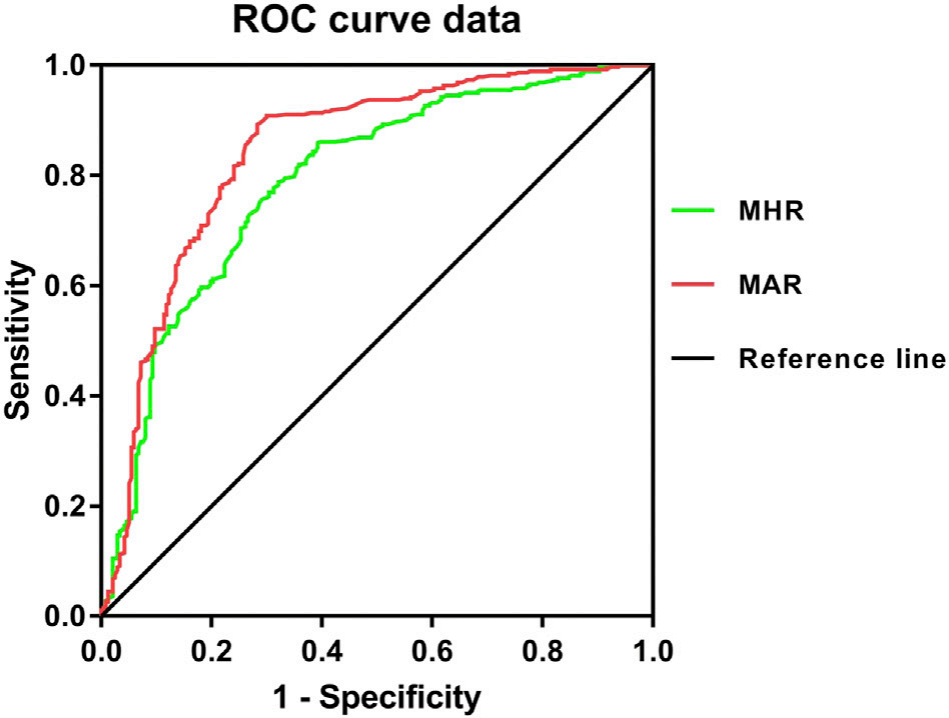
Receiver operating characteristic curves for the cutoff value of MHR and MAR identifying osteoporosis.

From the ROC curve analysis, the results seemed to have a good identifying value of MHR and MAR for osteoporosis. The AUC of MHR and MAR identifying osteoporosis were 0.791 (95% CI:0.753–0.828, *p* < 0.001) and 0.843 (95%CI: 0.809–0.877, *p* < 0.001) respectively (Figure 3). The optimal cut-off values of MHR and MAR were 4.53 × 10^8^/mmol (sensitivity: 60.8%, specificity: 85.9%) and 4.74 × 10^8^/g (sensitivity: 71.7%, specificity: 89.3%) respectively (Table 6).

**TABLE 6:** ROC Curve Analysis of MHR and MAR in identifying osteoporosis.

Variables	AUC(95% CI)	Cut-off value	Sensitivity (%)	Specificity (%)
MHR (10^8^/mmol)	0.791 (0.753–0.828)	4.53	60.8	85.9
MAR (10^8^/g)	0.843 (0.809–0.877)	4.74	71.7	89.3

MHR, monocyte to HDL-c ratio. MAR, monocyte to ApoA1 ratio.

## Discussion

Osteoporosis and T2DM have became major public health concerns in increasingly aging population, both of them are closely associated with severe morbidity and increased mortality. As the main risk factor of osteoporosis and bone fragility fractures, chronic low inflammation caused by T2DM can directly influence bone homeostasis. In this cross-sectional study, we mainly assessed the correlations between pro-inflammatory indexes of MHR, MAR and bone turnover markers, BMD in Chinese postmenopausal women with T2DM. The results in our study showed that MHR and MAR were positively associated with *β*-CTX and negatively associated with L1-L4 and femoral neck BMD after adjustment for other confounding factors. Moreover, MHR and MAR were also independently associated with higher odds of osteoporosis and seemed to have a good identifying value for osteoporosis.

Emerging evidences showed that chronic metabolic disorders can trigger inflammatory responses as a mechanism for coping with metabolic changes, leading to chronic inflammation occurred (Zhang et al., 2012). Clinical studies also observed a concurrent increase in circulating inflammatory cytokines in T2DM. The persistent hyperglycaemia can alter the release and functionality of inflammatory cytokines like interleukin 1 (IL-1), interleukin 6 (IL-6) and tumour necrosis factor-a (TNF-a) (Degirmenci et al., 2019), leading to an increase in T2DM. In addition, all these cytokines can increase the risk of osteoporosis and bone fragility fractures, and mediate certain aspects of bone physiology *via* nuclear factor kappa-light-chain enhancer of activated B cells pathway (Liu and Zhang, 2015). In the process of chronic inflammation, monocyte play an important role in initiating and up-regulating inflammatory responses. Monocyte are produced from bone marrow and accumulated in circulatory system, it can stimulate the immune system and increase inflammatory responses by releasing inflammatory cytokines like TNF-ɑ, IL-6 and monocyte chemoattractant protein-1 (Ghanim et al., 2004; Orozco Susana et al., 2021). Clinical studies also observed that inflammatory and hyperglycemic environment caused by T2DM can increase the peripheral total monocyte counts compared with healthy population (Tsai Jack et al., 2007; Gonzalez et al., 2012). Bala MM et al. enrolled 112 participants with obesity found that circulating monocyte count is negatively correlated with BMD in children and adolescents with obesity (Bala and Bala, 2022). These biological features and clinical findings provide a basis for monocyte to be a ideal inflammatory marker component for chronic inflammation caused by T2DM.

Dyslipidemia is another kind of metabolic disorders occurred in T2DM with increased LDL-c and decreased HDL-c and its major apolipoproteins (APOA1). The decrease of HDL-c and its major apolipoproteins (APOA1) was also reported to be associated with increased oxidative stress and systemic inflammation. HDL-c and APOA1 are clusters of “good cholesterol” can bind lipid molecules and participate in the process of cholesterol clearance. Both of them were also thought to have anti-oxidant and anti-inflammatory properties. Recent advances in lipoprotein highlighted the important roles of HDL-c in bone metabolism. HDL-c and its major apolipoproteins (APOA1) were reported to have a direct interaction effect on osteoblasts and osteoclasts in experimental mouse models (Triantaphyllidou et al., 2013). Clinical studies also found that HDL-c can directly interact with osteoblasts and osteoclasts, whereas there is no single consensus has been reached on the correlation between HDL-c and BMD, and this correlation may vary by race and hormonal status. The vast majority of studies in Asian postmenopausal women reported that HDL-c is positively associated with lumbar spine BMD (Yamaguchi et al., 2002; Jeon Yun et al., 2011; Li et al., 2015). It can be assumed that the MHR and MAR may be ideal inflammatory markers to reflect bone homeostasis imbalance caused by chronic inflammation in bone micro-environment.

β-CTX is a fragment of medium collagen degraded and released into the blood during bone remodeling, it can reflect the degree of bone resorption and bone loss, it has been considered as a marker of increased osteoclast activity. Most studies reported chronic inflammation in bone micro-environment can enhance osteoclast activity, leading to an increase in *β*-CTX (Eleftheriadis et al., 2008). In present study, the results confirmed our hypothesis, MHR and MAR were positively associated with *β*-CTX after adjustment for other confounding factors. Despite the BMD T-score may underestimate fracture risk in T2DM, while the detection of BMD by DXA does predict fracture risk in clinical practice (Schwartz et al., 2011). Previous studies reported an increase risk of vertebral and femoral neck fractures in T2DM. In our study, we also found that MHR and MAR were negatively associated with L1-L4 and femoral neck BMD. These findings may indicate us that MHR and MAR can reflect bone homeostasis imbalance caused by chronic inflammation. Clinical studies observed MHR and MAR have good predictive values for chronic low inflammatory diseases like peripheral artery disease (Selvaggio et al., 2020), polycystic ovarian syndrome (Kałużna et al., 2020),nonalcoholic fatty liver disease (Wang et al., 2022), metabolic syndrome (Uslu Ali et al., 2018), central retinal artery occlusion (Guven and Kilic, 2021), unstable angina (Zhang et al., 2016) and parkinson’s disease (Liu et al., 2021). To our surprise, the results in our study also found that MHR and MAR were not only a independent risk factor of osteoporosis, they were also seemed to have a good identifying value for osteoporosis with relatively high AUC values of 0.791 and 0.843. MHR and MAR may be potential indicators of osteoporosis for Chinese postmenopausal women with T2DM, whereas more longitudinal studies are need to further confirm these findings.

To our knowledge, our study firstly put insights into the associations between pro-inflammatory indexes (MHR and MAR) and bone turnover markers, BMD in Chinese postmenopausal women with T2DM. Meanwhile, some limitations are need to be mentioned. Despite this study adjusted several potential confounding variables in final analysis and included enough sample size can represent the Chinese newly diagnosed T2DM population, while this study was designed as a cross-sectional study without follow up, it can not directly reflect the associations between MHR, MAR and bone turnover markers, BMD. Second, due to the study population are Chinese postmenopausal women with T2DM, the associations in our studies may be not applicable to other races and hormonal status.

In conclusion, this study found that novel pro-inflammatory indexes of MHR and MAR were associated with bone resorption marker of *β*-CTX,L1-L4 and femoral neck BMD. In addition, MHR and MAR were also independently associated with osteoporosis and seemed to have a good identifying value for osteoporosis. These findings may indicate us MHR and MAR are ideal pro-inflammatory markers to reflect bone homeostasis imbalance caused by chronic inflammation in bone micro-environment in Chinese postmenopausal women with T2DM, whereas more studies with enough follow-up are need to further evaluate these findings and illustrate the underlying mechanisms.

## Data Availability

The original contributions presented in the study are included in the article/Supplementary Material, further inquiries can be directed to the corresponding author.
